# The effect of a postpartum IUD intervention on counseling and choice: Evidence from a cluster-randomized stepped-wedge trial in Sri Lanka

**DOI:** 10.1186/s13063-019-3473-6

**Published:** 2019-07-08

**Authors:** Mahesh Karra, Erin Pearson, Elina Pradhan, Ranjith de Silva, Arnjali Samarasekera, David Canning, Iqbal Shah, Deepal Weerasekera, Hemantha Senanayake

**Affiliations:** 1 Frederick S. Pardee School of Global Studies, Boston University, 121 Bay State Road, Boston, MA 02215 USA; 2 Harvard T.H. Chan School of Public Health, Boston, MA 02115 USA; 3 Ipas, Chapel Hill, NC 27515 USA; 4 World Bank Group, Washington, DC 20006 USA; 5 Sri Lanka College of Obstetricians and Gynaecologists, Colombo, 08 Sri Lanka

**Keywords:** Immediate postpartum IUD (PPIUD), Family planning, FIGO, Intervention, Counseling, Uptake, 44 cluster-randomized controlled trial, Stepped-wedge design, Sri Lanka

## Abstract

**Background:**

The International Federation of Gynaecology and Obstetrics (FIGO), in collaboration with the Sri Lankan College of Obstetrics and Gynaecologists (SLCOG), launched an initiative in 2014 to institutionalize immediate postpartum IUD (PPIUD) services as a routine part of antenatal counseling and delivery room services in Sri Lanka. In this study, we evaluate the effect of the FIGO-SLCOG PPIUD intervention in six hospitals by means of a cluster-randomized stepped-wedge trial.

**Methods/design:**

Six hospitals were randomized into two groups of three using matched pairs. Following a 3-month baseline period, the intervention was administered to the first group, while the second group received the intervention after 9 months of baseline data collection. We collected data from 39,084 women who delivered in these hospitals between September 2015 and January 2017. We conduct an intent-to-treat (ITT) analysis to determine the impact of the intervention on PPIUD counseling and choice of PPIUD, as measured by consent to receive a PPIUD, as well as PPIUD uptake (insertion following delivery). We also investigate how factors related to counseling, such as counseling timing and quality, are linked to choice of PPIUD.

**Results:**

We find that the intervention increased rates of counseling, from an average counseling rate of 12% in all hospitals prior to the intervention to an average rate of 51% in all hospitals after the rollout of the intervention (0.307; 95% CI 0.148–0.465). In contrast, we find the impact of the intervention on choice of PPIUD to be less robust and mixed, with 4.1% of women choosing PPIUD prior to the intervention compared to 9.8% of women choosing PPIUD after the rollout of the intervention (0.027; 95% CI 0.000–0.054).

**Conclusions:**

This study demonstrates that incorporating PPIUD services into postpartum care is feasible and potentially effective. Taking the evidence on both counseling and choice of PPIUD together, we find that the intervention had a generally positive impact on receipt of PPIUD counseling and, to a lesser degree, on choice of the PPIUD. Nevertheless, it is clear that the intervention’s effectiveness can be improved to be able to meet the demand for postpartum family planning of women.

**Trial registration:**

ClinicalTrials.gov, NCT02718222. Registered on 11 March 2016 (retrospectively registered).

**Electronic supplementary material:**

The online version of this article (10.1186/s13063-019-3473-6) contains supplementary material, which is available to authorized users.

## Background

The World Health Organization (WHO) recommends that a woman wait at least 24 months after a live birth before attempting the next pregnancy in order to reduce the risk of adverse maternal and child health outcomes [1, 2]. However, a woman’s fertility may return as soon as 4 weeks after delivery, particularly if she is not breastfeeding [3]. Given that most women are unaware of how soon fertility can return postpartum, they may not initiate contraception in a timely fashion and may be at greater risk of unintended pregnancy. In 17 of the 43 countries with a Demographic and Health Survey (DHS) from 2005 to 2013, the provision of postpartum family planning (PPFP) and long-acting reversible methods of contraception to women who wanted no more children was especially rare [4]. Studies have also shown that many women who expressed a desire to postpone or limit childbearing do not receive any postpartum method because they fail to return for a postnatal visit, are lost to follow up, or face other barriers to receiving care [5, 6]. As a result, two out of three women are estimated to have an unmet need for contraception in the year following the birth of a child [7].

The provision of effective postpartum contraception, particularly long-acting reversible methods such as the copper intrauterine device (IUD), has been shown to reduce the risk of pregnancy and is associated with higher continuation of use than other methods 6 months following a delivery [8]. The immediate postpartum IUD (PPIUD) is a long-acting, reversible method of contraception that can be used safely and effectively within 48 h of delivery and even while breastfeeding [9]. The PPIUD offers a convenient contraceptive option to women who cannot return for follow-up visits because of distance, travel costs, and time constraints, or other barriers to access. Studies have shown that with adequate and effective provider training, expulsion and complication rates of PPIUD insertions are similar to those of interval IUDs, which are inserted 4 to 6 weeks after delivery [9–12].

The International Federation of Gynaecology and Obstetrics (FIGO), in collaboration with its national affiliate in Sri Lanka, the Sri Lankan College of Obstetrics and Gynaecologists (SLCOG), launched an initiative in 2014 to institutionalize PPIUD services as a routine part of antenatal counseling and delivery room services in Sri Lanka. This intervention included: a) training of health care providers to counsel women in PPFP during antenatal care visits and in hospitals; b) training of doctors in the insertion of the PPIUD, ensuring the provision of required supplies for PPIUD insertion in delivery rooms; and c) follow up of women who chose PPIUD. Similar initiatives were also launched in five other countries: Nepal, Tanzania, Kenya, Bangladesh, and India.

In this study, we evaluate the effect of the FIGO-SLCOG PPIUD intervention on PPIUD counseling and choice of PPIUD in six hospitals in Sri Lanka by means of a cluster-randomized stepped-wedge trial. As part of the trial design, the six hospitals were randomized into two groups of three. Following a 3-month baseline period, the intervention was administered to hospitals assigned to the first group, while hospitals in the second group received the intervention after 9 months of baseline data collection—consequently, these hospitals started the intervention 6 months after the first group of hospitals had started the intervention. A similar cluster-randomized stepped-wedge design to evaluate the impact of the FIGO intervention was also implemented in Nepal and Tanzania.

We collected data from women who delivered in these six study hospitals between September 2015 and January 2017, and we conduct an ITT analysis to determine the impact of the intervention on counseling and choice of PPIUD. As part of this analysis, we also investigate how factors related to receipt of counseling, such as counseling timing and quality, are linked to choice of PPIUD, as measured by consent to receive a PPIUD as well as PPIUD uptake.

### The FIGO-SLCOG PPIUD intervention

The PPIUD intervention program in Sri Lanka was developed by FIGO in collaboration with SLCOG and with support from the Family Health Bureau (FHB), which is the primary organization within the Ministry of Health that is responsible for oversight, coordination, and evaluation of reproductive health and maternal and child health programs in Sri Lanka. The intervention program in Sri Lanka was rolled out to 18 hospitals in three waves. Each wave consisted of six hospitals receiving the intervention. Findings using monitoring data from the first wave of six hospitals that received the intervention are presented elsewhere [13]. This study uses data from the third wave of six hospitals, which were the only wave of hospitals where the intervention was rolled out in a staggered stepped-wedge cluster-randomized study design described above.

The intervention was designed to adhere to the national guidelines for the provision of family planning services and to ensure sustainability of any future scale-up of the program. The intervention included: (1) workshops on PPFP and PPIUD for doctors, midwives, nurses, and general hospital staff who worked in maternity wards; (2) the training of maternity care providers in hospitals and in surrounding Ministry of Health (MOH) antenatal clinics in PPFP counseling; (3) the training of doctors in study hospitals in PPIUD insertion; (4) the provision of PPFP leaflets to hospitals and MOH clinics to be distributed during counseling; (5) the provision of a video on PPFP to be displayed in the hospital waiting area; (6) the provision of Kelley’s forceps for vaginal PPIUD insertion and of copper-T IUDs to hospitals; and (7) monitoring and evaluation of counseling activities and PPIUD insertions by SLCOG and FIGO.

Health professionals from study hospitals and surrounding MOH clinics who oversaw maternal and child health care were trained to provide counseling services during antenatal visits. Doctors who provided obstetric services in maternity wards were trained on PPIUD insertion and removal using the Kelley’s forceps and thread retrievers. Each workshop was one day long and covered topics related to counseling along with lectures and videos on the PPIUD and PPFP, more generally. Obstetric trainees were given opportunities to practice IUD insertion and removal on MAMA-U mannequin models for vaginal and intra-Caesarean procedures and were trained on infection prevention, side effects, and complication management. Pre-training and mid-training knowledge assessments were conducted along with role-plays and group discussions to facilitate the training. Finally, providers were also trained on how to disseminate PPFP leaflets, which include information about the benefits of birth spacing and contraceptive methods, during counseling with women.

## Methods/design

The protocol for the trial, which was implemented in Sri Lanka, Nepal, and Tanzania, has been registered with ClinicalTrials.gov (NCT02718222) and has been published elsewhere [14]. Although the protocol and study were retrospectively registered with ClinicalTrials.gov, the protocol did not change from initiation of the study until study protocol registration in Sri Lanka. The components of the study as they pertain to the analysis are described below.

A Data and Safety Monitoring Board (DSMB), which was comprised of representatives from FIGO, SLCOG, and Harvard University, met every 4 months to review interim results and monitor compliance with the study protocol and review any adverse events associated with the intervention, such as higher than expected expulsion or complication rates. Additional information can be found in the terms of reference for the PPIUD study DSMBs, which are available upon request. Findings on complications related to PPIUD insertion across all six intervention countries are presented elsewhere [12].

### Study design

A stepped-wedge cluster-randomized design was implemented to evaluate the intervention in six tertiary hospitals in Sri Lanka: Nuwara Eliya District General Hospital, Nawalapitiya District General Hospital, Polonnaruwa District General Hospital, Chilaw District General Hospital, Moneragala District General Hospital, and Kalutara District General Hospital. Four of the six hospitals (Polonnaruwa, Moneragala, Kalutara, and Chilaw) served mostly Sinhala-speaking women, while the other two hospitals (Nuwara Eliya and Nawalapitiya) served mostly Tamil-speaking women. As part of the randomization protocol, the six study hospitals were first matched into pairs based on similar geography, ethnolinguistic composition, and annual obstetric caseload. The hospitals were matched into pairs as follows: (i) Nawalapitiya and Nuwara Eliya, (ii) Kalutara and Chilaw, and (iii) Moneragala and Polonnaruwa. Following the paired matching, one hospital within each pair was randomized into the Group 1 (early intervention) treatment arm, while the other hospital in the pair was assigned to the Group 2 (late intervention) treatment arm: Nawalapitiya, Polonnaruwa, and Chilaw hospitals were randomized into the Group 1 arm, while Nuwara Eliya, Kalutara, and Moneragala hospitals were randomized into the Group 2 arm. Additional file 1: Figure S1 presents a map of the six hospitals according to their treatment group assignment.

Baseline data collection commenced on 7 September 2015. The three hospitals in Group 1 were scheduled to receive the training in mid-November 2015, and the implementation of the intervention would commence from 7 December 2015, 3 months after the start of baseline data collection.

Group 2 hospital staff and service providers were scheduled to receive the training in mid-June 2016 (with the intervention beginning from 7 July 2016), 9 months after the start of baseline data collection and 6 months after the rollout of the intervention in Group 1 hospitals, with the idea that the staggered rollout of the intervention would allow for causal comparisons between groups within the 6-month window. Given delays in the training implementation to hospital staff and the fact that intervention trainers needed time to travel between hospitals to administer the workshop, the actual timing of the intervention rollout in each hospital varied from the planned rollout date. To this end, Group 1 hospitals began the intervention a few weeks apart from each other, in December 2015, whereas the intervention in Group 2 began in July 2016.

### PPIUD activities in Nawalapitiya and Nuwara Eliya Hospitals

Following randomization of hospitals into Group 1 and Group 2 hospitals, it was discovered that PPIUD services were already being conducted in Nawalapitiya (a Group 1 hospital) and Nuwara Eliya (a Group 2 hospital) independently of and prior to the rollout of the FIGO-SLCOG intervention. In contrast to the FIGO-SLCOG protocols, senior medical faculty in Nawalapitiya had developed a method to insert PPIUDs using plastic inserters that were provided with standard IUD packs (as opposed to the longer, 32-cm curved Kelley’s forceps) under ultrasound guidance, while PPIUD insertion services in Nuwara Eliya were only offered to those women who delivered via cesarean section. In order to disentangle the impact of the FIGO-SLCOG intervention from these competing existing practices, the following measures were implemented:

1. Data on the type of forceps that were used for the insertion were collected.
2. Data on whether an ultrasound was used to guide the insertion were collected.
3. All of our main analyses use the full sample of six hospitals; however, we also re-ran the analyses on the sub-sample of women from the four hospitals where no PPIUD services were offered prior to the FIGO-SLCOG intervention. Results from the re-run analyses are presented in Additional file 1.

### Data collection

Five to six enumerators were posted in each study hospital and were trained to interview women in postnatal wards using a structured survey that was programmed onto handheld tablets. Women were recruited in postnatal wards following delivery. No compensation was provided to women who chose to participate in the interview, and no compensation was provided to women who chose to receive PPIUD insertions. The survey collected information on women’s sociodemographic background characteristics, birth histories, antenatal care for recent births, history of contraceptive use, receipt of family planning and PPIUD counseling during the antenatal and postnatal periods, satisfaction with any family planning counseling services that they might have received, choice and uptake of PPIUD, and fertility intentions.

### Outcomes of interest and key treatment variable

Our first outcome of interest is a measure of a woman’s receipt of PPIUD counseling. A woman in our study could have been counseled on PPIUD at one or more of the following times over the course of her pregnancy: 1) during an antenatal care visit to the hospital or at one of the hospital’s satellite MOH clinics; 2) at one of the hospital’s antenatal wards if she arrived early for the birth and was not in active labor; or 3) during postnatal care in the hospital ward following delivery. For our analysis, we construct a binary variable for receipt of PPIUD counseling that reflects a woman’s reported receipt of counseling either during an antenatal clinic visit or after admission to the hospital for delivery.

Our second outcome of interest is a measure of a woman’s choice to have a PPIUD inserted following her delivery. If a woman chose to have a PPIUD inserted, consent for insertion was taken either during her antenatal care visit or during her postnatal care in the hospital ward following delivery. Consent was confirmed and noted in maternity records before the PPIUD was inserted. For our analysis, we define PPIUD uptake to be an indicator variable for women who had a PPIUD insertion at any time. There were 148 women in our study who reported that they had consented to PPIUD as a postpartum contraceptive method but did not have a PPIUD inserted due to: a) complications during delivery and/or at the time of the insertion (22 women); b) consent not being confirmed because a written consent form was not available (one woman); or c) no PPIUD insertions were being performed in the ward at the time of their request (125 women). Given our intention to measure demand for PPIUD because of the intervention, we consider these women to have chosen to have a PPIUD inserted.

Our key treatment variable is a woman’s exposure to the intervention at the time of her delivery, which is based on whether she delivered in a hospital after the start of the intervention. A timeline of the intervention rollout in each hospital is presented in Additional file 1: Table S1.

### Analytic sample

Data were collected for women who delivered in the six hospitals in Sri Lanka between 7 September 2015 and 7 January 2017. All women who gave birth in these six hospitals over this period were eligible to be in the study sample unless their primary residence was outside of Sri Lanka. Out of 40,382 women who were admitted to the six hospitals over the 16-month enrolment period, 40,352 (99.9%) women were eligible for the study and 39,772 (98.5%) of eligible women consented to be interviewed. Interviews were conducted with women in hospital postnatal wards before women were discharged from the hospital following their delivery. After dropping observations with incomplete information on the outcome and covariate variables of interest, we obtain our analytic sample of 39,084 women (96.8% of admitted women) for the study.

### Analytic strategy

Our ITT analysis estimates the causal effect of the intervention on women’s receipt of PPIUD counseling and on women’s choice of PPIUD. This analysis employs a linear probability model of the outcome of interest (whether a woman was counseled, whether a woman chose a PPIUD) on women’s exposure to the intervention. Differences between hospitals and underlying trends over time are controlled for through hospital fixed effects and month fixed effects. The differential timing of the intervention across hospitals allows us to identify the causal effect of the intervention. We provide unadjusted estimates that only include hospital and month fixed effects as well as adjusted estimates that include additional covariates that capture background characteristics of the women. Woman-level covariates include: age at the time of delivery (in five-year age groups); educational attainment (none, primary, secondary, higher); ethnicity; parity; and the time taken to travel from the woman’s residence to the hospital (less than 1 h, between 1 to 3 h, and more than 3 h).

While the outcome variables are binary, we have a fully saturated model with discrete explanatory variables, where every individual is in one of a finite number of strata. In this case, the prediction of the outcome given by a linear probability model is simply the average outcome for the stratum, and hence is a well-specified model for the outcome. We can therefore estimate the ITT effect using a simple linear regression where the treatment effect is simply the difference in outcomes between the treatment and control groups [15].

Our main ITT regression is specified as follows:

1 $$ {\mathrm{Y}}_{\mathrm{iht}}=\upalpha +\upbeta {\mathrm{Post}}_{\mathrm{h}\mathrm{t}}+{\mathbf{X}}_{\mathbf{i}\boldsymbol{\upgamma }}+{\updelta}_{\mathrm{h}}+{\uptau}_{\mathrm{m}}+{\upvarepsilon}_{\mathrm{iht}} $$

Where the ***X***_*i*_ and γ are bolded because they are vectors. The dependent variable *Y*_*iht*_ is a dummy variable indicating whether woman *i* who gave birth in hospital *h*, on day *t* received the PPIUD outcome of interest (received PPIUD counseling or chose a PPIUD). The main explanatory variable of interest is ***Post***_*ht*_, which is an indicator variable that takes the value one in hospital *h* on days *t* after the start of the intervention and zero before the start of the intervention. Hospital fixed effects are captured by the term δ_*h*_, while *τ*_*m*_ represents month fixed effects for the 16 months of the study. The term ***X***_*i*_ is a vector of covariates that captures the characteristics of woman *i*.

The coefficient *β* captures the effect of the intervention. However, not all women in the intervention hospitals were counseled. Women may not have visited either the hospitals or one of the satellite MOH clinics, where providers were trained in PPIUD counseling, to receive antenatal care and instead may have chosen to receive care at local clinics nearer to their homes. As a result, they may not have been exposed to the PPIUD intervention until their arrival to the hospital for delivery. To account for this imperfect exposure to the intervention, we also estimate the adherence-adjusted effect of PPIUD counseling on choice of PPIUD, which captures the causal impact of being counseled on PPIUD due to exposure to the intervention on a woman’s likelihood of choosing PPIUD. Typically, an adherence-adjusted estimate is calculated using an instrumental variables (IV) approach, where exposure to the intervention would serve as an instrument for being counseled on PPIUD [16]. However, since our dependent variable (choice of PPIUD), instrument (exposure to the intervention), and explanatory variable that is being instrumented (counseled on PPIUD) are all binary, the standard IV approach for dealing with endogeneity is not technically specified and should be modified [17, 18]. To this end, we estimate the adherence-adjusted impact of PPIUD counseling on choice of PPIUD using a control function approach, which is a complementary estimation strategy to the IV and overcomes the specification problem that IV faces in models where the endogenous variable being instrumented is non-linear [19]. We present estimates of the adherence-adjusted impact from a linear probability model that, due to its linear specification, gives us identical estimates to the standard IV.

Finally, we present results from an analysis on how the quality of counseling affects choice of PPIUD among women who were counseled. We assess how being given a PPFP leaflet, having an opportunity to ask questions during a counseling visit, and knowledge of benefits or disadvantages of PPIUD are related to a woman’s likelihood of choosing PPIUD.

In all of our models, the outcomes for women who deliver in the same hospital are likely to be correlated; as a result, the standard errors of our estimates need to be corrected. Since we only have six hospitals, or six clusters, the standard cluster-robust variance estimator, which is an appropriate correction for clustered data where there are a large number of clusters, may be invalid [20]. We therefore use the wild cluster bootstrap method with a six-point weight distribution to generate corrected standard errors for our point estimates for all models. The use of the wild cluster bootstrap method for the correction of standard errors has been shown to have good properties with a small number of clusters [21–23].

## Results

Table 1 presents descriptive statistics of the analytic sample. Column 1 presents the mean of each variable for the full analytic sample of 39,084 women who delivered over the 16-month study period. Columns 2 and 3 present the mean of each variable for the sample of women in Group 1 and Group 2 hospitals who delivered during the first 3 months of data collection, before the intervention was rolled out to either group. With individual-level randomization of the intervention, we would expect there to be balance across covariates between treatment and control groups at baseline. However, since randomization was conducted at the hospital level, and given that there are only six hospitals, systematic differences between Group 1 and Group 2 hospitals are more likely. We therefore test the hypothesis that the two groups of hospitals have the same mean characteristics for women during the pre-intervention period using the wild bootstrap method (Column 4). Apart from minor differences in women’s educational attainment at baseline (Group 2 hospitals had a slightly higher proportion of women with secondary education), we find that most covariates are balanced across the two groups.

**Table 1:** Descriptive statistics for the full sample and during the first 3 months of baseline, by group

	Full sample	Group 1 baseline mean	Group 2 baseline mean	Difference (Group 2 mean − Group 1 mean)^1^
		First 3 months	First 3 months	First 3 months
Panel A				
Woman’s age				
< 20 years	0.051	0.049	0.051	0.002
20–24 years	0.232	0.227	0.226	− 0.001
25–29 years	0.325	0.32	0.315	− 0.005
≥ 30 years	0.393	0.404	0.408	0.004
Woman’s schooling				
No schooling	0.007	0.007	0.009	0.002
Some primary	0.111	0.119	0.119	0
Some lower secondary	0.375	0.247	0.365	0.118*
Some higher secondary	0.247	0.355	0.277	− 0.078
Some college	0.259	0.272	0.23	− 0.042
Time taken to travel from home to hospital				
< 1 h	0.505	0.575	0.476	− 0.099
1–3 h	0.44	0.375	0.451	0.076
≥ 3 h	0.055	0.05	0.073	0.023
Parity				
1	0.385	0.378	0.369	− 0.009
2	0.377	0.377	0.373	− 0.004
3+	0.238	0.245	0.257	0.013
Ethnicity				
Sinhalese	0.708	0.761	0.695	− 0.066
Sri Lankan Tamil	0.105	0.097	0.107	0.01
Indian Tamil	0.084	0.047	0.1	0.053
Sri Lankan Moor	0.1	0.093	0.091	− 0.002
Other	0.003	0.001	0.006	0.005
Male child born	0.513	0.507	0.518	0.011
Received ANC				
Hospital	0.472	0.477	0.464	− 0.013
MOH clinic	0.817	0.936	0.738	− 0.198
Panel B				
Received PPIUD counseling	0.349	0.233	0.079	− 0.153
Choice of PPIUD	0.07	0.127	0.015	− 0.112
N	39,084	3478	4180	

***p < 0.01, **p < 0.05, *p < 0.1

^1^Significance of difference tested using wild-cluster bootstrap method

Note: Balance table across baseline and intervention period shown in Additional file 1: Table S2. Balance table across baseline and intervention period in each hospital is shown in Additional file 1: Table S3

**Fig. 1:**
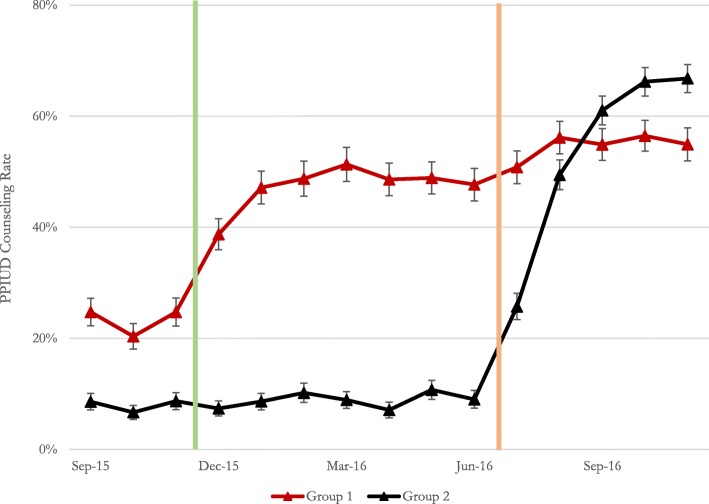
Trends in PPIUD counseling rates standard errors are shown as *error bars*. Approximate intervention start dates in Group 1 (*red*) and Group 2 (*black*) hospitals are shown by the *green* and *orange vertical lines*, respectively. For exact dates of intervention, please see Additional file 1: Table S1

Figure 1 presents the trend in PPIUD counseling rates by the two groups over the study period. During the baseline period, the four hospitals with no prior PPIUD history (Polonnaruwa and Chilaw from Group 1, and Moneragala and Kalutara from Group 2) had very low counseling rates (see Additional file 1: Table S4). On the other hand, over 85% of women were counseled on PPIUD in Nawalapitiya hospital (a Group 1 hospital) over this time, while between 22 and 29% of women who came to Nuwara Eliya hospital (a Group 2 hospital) for delivery were counseled on PPIUD. In examining the average group counseling rates over this period, we see that between 20 and 24% of women in Group 1 hospitals were counseled on PPIUD (the vast majority of whom were counseled at Nawalapitiya hospital), while between 6 and 8% of women in Group 2 hospitals were counseled on PPIUD (the vast majority of whom were counseled at Nuwara Eliya hospital).

There was a clear rise in counseling rates in both groups of hospitals immediately after the start of the PPIUD intervention. Interestingly, the rate of increase in counseling after the start of intervention in Group 2 hospitals was generally higher than the rate of increase in counseling in Group 1 hospitals, whereby a larger proportion of women from Group 2 hospitals were counseled than women from Group 1 hospitals by the end of the study period.

Table 2 presents statistics of the timing of PPIUD counseling sessions and of measures of counseling quality among women who reported being counseled. The majority of women who were counseled on PPIUD were counseled in the antenatal period (64.7%), while only 8.9% of counseled women reported to have received counseling only after admission for delivery; 26.4% of counseled women reported receiving counseling both before and after admission. Among counseled women, 59% reported having been given an opportunity to ask questions during counseling; however, only 24.3% of women reported having received the PPFP leaflet. As a means of testing counseling quality, women who were counseled were asked to recall some benefits and some disadvantages of the PPIUD that counselors were trained to discuss with them during their counseling session. Enumerators checked these responses against a list of benefits and disadvantages. Among those counseled, 68.5% of women could only recall the benefits of receiving a PPIUD, while only 12.3% of women could recall at least one benefit and one disadvantage; 19.2% of women could either not recall any benefits or disadvantages of the method or could only recall disadvantages of the method.

**Table 2:** Characteristics of counseling for PPIUD and PPIUD knowledge if counseled

n (proportion)	
PPIUD knowledge	
Can’t recall any benefits/disadvantages, or recall disadvantages only	2607 (0.192)
Recall benefit(s) only	9289 (0.685)
Recall both benefit(s) and disadvantage(s)	1666 (0.123)
Timing of PPIUD counseling	
Before admission, during ANC	8824 (0.647)
After admission only	1218 (0.089)
Both	3606 (0.264)
Women given opportunity to ask questions	8009 (0.59)
Woman given a leaflet during counseling	3302 (0.243)
Total	13,569 (1.000)

**Fig. 2:**
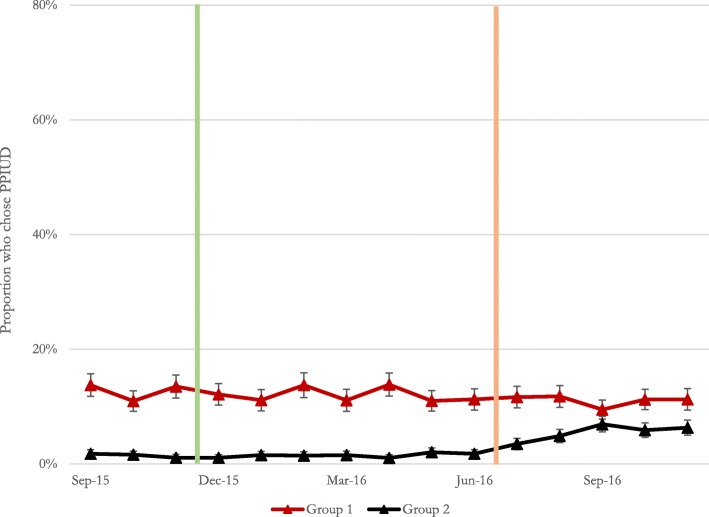
Trends in choice of PPIUD. Standard errors are shown as *error bars*. Approximate intervention start dates in Group 1 (*red*) and Group 2 (*black*) hospitals are shown by the *green* and *orange vertical lines*, respectively. For exact dates of intervention, please see Additional file 1: Table S1

Figure 2 presents results for women’s choice of PPIUD. During the baseline period, choice of PPIUD was found to be very low or non-existent in all hospitals except for Nawalapitiya and Nuwara Eliya hospitals, where PPIUD services were being provided prior to the rollout of the intervention. In Nawalapitiya hospital, choice of PPIUD was higher than all other hospitals combined throughout the entire study period, with rates as high as 50% in some months. Choice of PPIUD in Nuwara Eliya hospital was lower than in Nawalapitiya, ranging between 3 and 6%. In the four hospitals where PPIUD services were not available prior to the intervention, there was little evidence of any choice of PPIUD during the baseline period and modest evidence starting immediately after the intervention. However, there is high month-to-month variability in choice of PPIUD both across hospitals as well as within the same hospital over time. As was observed with PPIUD counseling, choice of PPIUD was higher in Group 2 hospitals than in Group 1 hospitals.

Table 3 presents estimates of the ITT effect of the intervention on receipt of PPIUD counseling. Columns 1 and 2 present estimates of the effect from the unadjusted model, while Columns 3 and 4 present estimates from the adjusted model. On average, we find that the intervention increased counseling by 30.7 percentage points (95% CI 14.8–46.5 pp). Our estimated effect does not depend on the inclusion of the control variables; there is little difference in the magnitude between the unadjusted and adjusted model estimates. This is not surprising since the added variables do not change significantly between the baseline and intervention periods and are therefore unlikely to explain the increase in the outcome between the two periods.

**Table 3:** Intent-to-treat effect of the intervention on PPIUD counseling

	Dependent variable: counseled on PPIUD			
	Est.	95% CI	Est.	95% CI
Post-treatment (ref: pre-treatment)	0.299***	0.159–0.439	0.306***	0.148–0.465
Woman’s age (ref: < 20 years)				
20–24 years			0.016	− 0.032–0.064
25–29 years			0.033	− 0.017–0.082
≥ 30 years			0.026	− 0.034–0.086
Woman’s Schooling (Ref: No schooling)				
Some primary			0.069	− 0.021–0.159
Some lower secondary			0.104**	0.013–0.195
Some higher secondary			0.124**	0.034–0.213
Some college			0.114**	0.016–0.213
Time to travel from home to hospital (ref: < 1 h)				
1–3 h			− 0.024	− 0.063–0.016
≥ 3 h			− 0.068***	− 0.107 - -0.030
Parity (ref: 1)				
2			0.022	−0.022–0.065
3+			− 0.000	− 0.018–0.017
Ethnicity (ref: Sinhalese)				
Sri Lankan Tamil			− 0.027	− 0.080–0.027
Indian Tamil			− 0.159**	− 0.305 - -0.013
Sri Lankan Moor			− 0.104**	− 0.200–− 0.008
Other			0.002	− 0.103–0.108
Male child born			− 0.008	− 0.020–0.004
Constant	0.117*	− 0.016–0.251	− 0.003	− 0.244–0.239
Observations	39,083		36,308	
R-squared	0.355		0.352	

***p < 0.01, **p < 0.05, * *p* < 0.1

Note: Difference from null tested using wild-cluster bootstrap method. All regression models adjusted for hospital and month fixed effects

While the intervention increased counseling rates overall, Table 3 also shows that some subgroups of women were more likely to be counseled. In particular, women with at least higher secondary education were more likely to receive PPIUD counseling. Indian Tamil and Sri Lankan Moor women were less likely to receive counseling, as were women who lived farther (more than 3 h) from the hospital. The heterogeneity of the effect of the intervention across these subgroups may reflect a lack of access to counseling services, particularly for women who live far from the hospital, and possibly indicate either a provider bias in determining the appropriateness of PPIUD to certain women or a hesitation or refusal by some women to be counseled on the method.

In Table 4, we report the ITT estimates of the effect of the intervention on choice of PPIUD. We find the intervention increased choice of PPIUD by 2.7 percentage points (95% CI 0.01–5.4 pp). In contrast to our findings on counseling, we observe that choice of PPIUD does not significantly vary by subgroups.

**Table 4:** Intent-to-treat effect of the intervention on choice of PPIUD

		Dependent variable: choice of PPIUD		
	Est.	95% CI	Est.	95% CI
Post-treatment (ref: pre-treatment)	0.024**	0.002–0.045	0.027**	0.000–0.054]
Woman’s age (ref: < 20 years)				
20–24 years			− 0.013	− 0.039–0.014
25–29 years			− 0.018	− 0.063–0.028
≥ 30 years Woman’s schooling (ref: no schooling)			− 0.016	− 0.071–0.039
Some primary			0.010	− 0.006–0.026
Some lower secondary			0.018	−0.024–0.059
Some higher secondary			0.012	−0.009–0.033
Some college			0.007	−0.012–0.027
Time to travel from home to hospital (ref: < 1 h)				
1–3 h			− 0.001	− 0.009–0.007
≥ 3 h			− 0.001	−0.009–0.006
Parity (ref: 1)				
2			0.026	− 0.016–0.068
3+			−0.019	− 0.073–0.035
Ethnicity (ref: Sinhalese)				
Sri Lankan Tamil			0.043	−0.090–0.176
Indian Tamil			0.058	−0.138–0.254
Sri Lankan Moor			−0.002	− 0.034–0.030
Other			0.054	− 0.071–0.178
Male child born			−0.002	− 0.006–0.002
Constant	0.021	− 0.019–0.062	0.178	− 0.224–0.580
Observations	39,084		36,309	
R-squared	0.253		0.253	

*** *p* < 0.01, ** p < 0.05, * *p* < 0.1

Note: Difference from null tested using wild-cluster bootstrap method. All regression models adjusted for hospital and month fixed effects

The ITT estimates in Table 4 present the impact of the intervention on choice of PPIUD. However, PPIUD counseling coverage was incomplete even during the intervention period, and many more women might have chosen PPIUD if they had been counseled. We therefore seek to estimate the causal effect of being counseled on choice of PPIUD, which would give us an estimate of how successful the intervention could have been if all women had been counseled. A direct estimate of the effect of counseling on PPIUD uptake is likely to be biased if counseling efforts by providers were targeted to focus on women who were more likely to choose PPIUD. In order to account for this potential targeted counseling, we estimate the “adherence adjusted” effect of the intervention. Typically, such an estimate would be computed using an IV approach, where the predicted probability of counseling from Column 3 of Table 3 would be used as the explanatory variable in a regression of choice on counseling in a two-stage procedure [16]. However, given the problems that the two-stage IV faces when both the explanatory variable and outcome are binary, we turn to a control function approach. The control function approach is very similar to the IV; however, it differs in its use of the residuals of the first stage estimation, rather than the predicted probabilities, as an additional explanatory variable in the second stage regression of choice of PPIUD on counseling. In the case where a linear model is run, the control function approach gives us identical estimates to a two-stage IV. Results of the estimated adherence-adjusted impact from this approach are presented in Table 5. We see that the receipt of counseling due to the intervention increases choice of PPIUD. Estimates from the model suggest an 8.9 percentage point (95% CI 2.7–15.0 pp) increase in choice of PPIUD from counseling all women.

**Table 5:** Adherence adjusted impact of PPIUD counseling on choice of PPIUD—a control function approach, linear probability model

	Choice of PPIUD			
	Est.	95% CI	Est.	95% CI
Counseled on PPIUD	0.079**	0.027–0.131	0.089**	0.028–0.150
Woman’s age (ref: < 20)				
20–24 years			− 0.014	− 0.038–0.010
25–29 years			− 0.021	− 0.063–0.022
≥ 30 years			− 0.018	− 0.068–0.032
Woman’s schooling (ref: no schooling)				
Some primary			0.004	−0.010–0.017
Some lower secondary			0.008	−0.026–0.042
Some higher secondary			0.001	−0.018–0.020
Some college			− 0.003	− 0.027–0.021
Time to travel from home to hospital (ref: < 1 h)				
1–3 h			0.001	−0.004–0.006
≥ 3 h			0.005	−0.003–0.012
Parity (ref: 1)				
2			0.024	−0.016–0.065
3+			−0.019	− 0.072–0.034
Ethnicity (ref: Sinhalese)				
Sri Lankan Tamil			0.046	−0.092–0.183
Indian Tamil			0.073	− 0.110–0.255
Sri Lankan Moor			0.007	− 0.024–0.039
Other			0.054	− 0.073–0.180
Male child born			− 0.002	− 0.006–0.003
Control function (FS Resids)	0.031	− 0.056–0.118	0.020	−0.058–0.098
Constant	0.012	− 0.016–0.041	0.178	−0.200–0.557
Observations	39,083		36,308	
R-squared	0.280		0.280	

***p < 0.01, **p < 0.05, *p < 0.1

Note: Differences from the null hypothesis are tested using the wild-cluster bootstrap method. Second stage results are shown above; first stage results are presented in Table 4. All regression models adjusted for hospital and month fixed effects

**Fig. 3:**
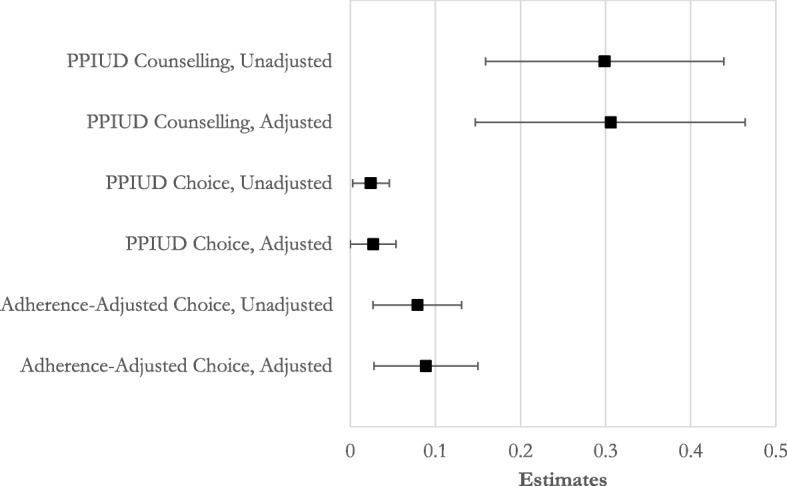
Forest plot of estimates from main analyses. 95% confidence intervals are shown as *error bars*

Figure 3 presents a Forest plot that summarizes all estimates (both adjusted and unadjusted) from the main analyses: counseling, choice of PPIUD, and the adherence-adjusted estimates of intervention impact. The graphical representation of the findings reinforces the robustness of the impact of the PPIUD intervention on these outcomes of interest.

Finally, Table 6 presents the determinants of choice of PPIUD among counseled women. Women who were counseled in the hospital after admission were more likely to choose a PPIUD. Measures of counseling quality (having the opportunity to ask questions during counseling, being able to remember benefits and disadvantages of PPIUD) are positively correlated with choosing a PPIUD. Indian Tamil women who were counseled were more likely to choose a PPIUD relative to counseled Sinhala women, and choice of PPIUD was higher among counseled women with two children relative to counseled women with one child. However, being given a PPIUD leaflet was not related to choosing PPIUD. Interestingly, counseled women with more schooling were less likely to choose a PPIUD relative to counseled women with less schooling.

**Table 6:** Determinants of choice of PPIUD among women who were counseled

	Dependent Variable: Choice of PPIUD	
	Est.	95% CI
Woman given a leaflet during counseling	0.040	−0.020–0.100
PPIUD knowledge (ref: Women can’t recall any benefits/disadvantages, or disadvantages only)		
Recall benefit(s) only	0.068**	0.007–0.129
Recall both benefit(s) and disadvantage(s)	0.065***	0.026–0.105
Women given opportunity to ask questions	0.061**	0.007–0.115
Timing of PPIUD counseling (ref: Before admission, during ANC)		
After admission only	0.280**	0.035–0.524
Both	0.327***	0.124–0.530
Woman’s age (ref: < 20)		
20–24 years	− 0.026	− 0.064–0.011
25–29 years	−0.043	− 0.107–0.021
≥ 30 years	−0.040	− 0.115–0.034
Woman’s schooling (ref: no schooling)		
Some primary	−0.038*	− 0.082–0.005
Some lower secondary	−0.041	− 0.110–0.028
Some higher secondary	− 0.055**	− 0.097–− 0.012
Some college	−0.069**	− 0.131–− 0.007
Time to travel from home to hospital (ref: < 1 h)		
1–3 h	−0.002	− 0.009–0.006
≥ 3 h	0.000	−0.019–0.019
Parity (ref: 1)		
2	0.036**	0.002–0.071
3+	−0.032	− 0.102–0.038
Ethnicity (ref: Sinhalese)		
Sri Lankan Tamil	0.078	−0.066–0.221
Indian Tamil	0.146	− 0.046–0.337
Sri Lankan Moor	0.007	− 0.060–0.074
Other	0.125	− 0.076–0.326
Male child born	− 0.004	− 0.014–0.006
Constant	0.337***	0.163–0.511
Observations	13,032	
R-squared	0.357	

***p < 0.01, **p < 0.05, *p < 0.1

Note: Difference from null tested using wild-cluster bootstrap method

Given the exceptional cases of Nawalapitiya and Nuwara Eliya hospitals in providing PPIUD services before the rollout of the intervention, we re-run the analysis excluding these two hospitals in order to more accurately measure the impact of the intervention on outcomes. Results from this analysis are presented in Additional file 1. In running this restricted analysis, we find that the ITT effects of the intervention on receipt of PPIUD counseling (Additional file 1: Table S6) are similar in magnitude to the estimates from the full sample. However, we no longer observe an impact of the intervention on choice of PPIUD. We find similar results when conducting the adherence-adjusted analysis for this subsample. Taken together, these results suggest that while the introduction of the intervention had an impact on counseling, the increase in choice of PPIUD over this study period is likely driven by a combination of established PPIUD activities along with the introduction of the intervention.

## Discussion

In this study, we evaluate the effect of the FIGO-SLCOG PPIUD intervention by means of a cluster-randomized stepped-wedge trial. Our results show that the intervention had an impact on increasing PPIUD counseling rates (0.307; 95% CI 0.148–0.465) and, to a less robust extent, choice of PPIUD among women in the six study hospitals (0.027; 95% CI 0.000–0.054). Receipt of counseling varied considerably across hospitals and within hospitals over the study period. Given the intervention’s focus on training providers in the hospital delivery wards and antenatal care facilities as well as in the hospitals’ satellite MOH clinics, a large proportion of women, particularly those living far from their hospital or MOH clinic, may have received their antenatal care elsewhere. There is substantial variation in counseling rates between different groups of women, which may reflect both variation in women’s access to hospital- and satellite clinic-based antenatal services as well as other underlying dynamics (e.g., provider bias in counseling for different groups, reluctance of women from certain groups to receive counseling, etc.). Our adherence-adjusted estimates suggest that if counseling had covered all women in the sample, choice of PPIUD would have increased by around 7 to 9 percentage points (0.089, 95% CI 0.028–0.150).

Rollout of insertion services also varied considerably over the study period and within the four hospitals with no prior history of providing PPIUD. For example, insertion services in Chilaw hospital were not provided for almost one year after the start of the intervention in the hospital due to delays in implementation, hospital administration changes, and frequent changes to PPIUD-trained medical staff (particularly medical residents in the hospital ward), who would be reassigned to other hospitals every 6 months. As a result, women who came to Chilaw hospital for delivery would be counseled on PPIUD during the antenatal period but would not receive insertion services at the hospital.

Among counseled women, women who knew about both the benefits and disadvantages of the PPIUD (0.065, 95% CI 0.026–0.105) as well as women who were given the opportunity to ask questions about the PPIUD (0.061, 95% CI 0.007–0.115) were more likely to take up the method. These findings suggest that improvements in counseling quality may serve to increase the number of women choosing the PPIUD as a contraceptive method. Counseled women who had two previous births were more likely to choose the PPIUD (0.036, 95% CI 0.002–0.071), which reflects the existing evidence on the positive correlation between parity and women’s desires to space or limit their future pregnancies [24]. These findings have implications for how these subgroups might be prioritized by policymakers when scaling up the intervention to reach populations with the highest demand for postpartum contraception. In contrast to the typically observed positive correlation between women’s educational attainment and contraceptive use, we find that counseled women with higher educational attainment were less likely to choose a PPIUD than counseled women with lower educational attainment (− 0.069, 95% CI − 0.131—− 0.007]. If we take women’s education as a marker for their socioeconomic status, our finding suggests that women who are more educated (and who may be better off socioeconomically) may have a wider range of postpartum contraceptive options outside of the PPIUD than women who are less educated (and who may be worse off socioeconomically).

Finally, we find differences in receipt of PPIUD counseling by ethnicity, particularly for minorities. These results may indicate either an underlying latent provider bias or reluctance to offer PPIUD services to certain types of women or a reluctance among minority women to approach providers for services.

When considering our sample, we note that the women in our study are not nationally representative; on average, they are younger and have considerably more years of schooling than women of reproductive age in Sri Lanka [25]. In targeting larger tertiary hospitals with high obstetric caseloads, our study, by design, excludes women who delivered either at home or at smaller primary health care facilities with more limited capacity for services. As of 2015, however, fewer than 1% of women in Sri Lanka delivered at home, and the vast majority of women (93%) delivered in facilities with access to a specialist obstetrician [26]. While it therefore may be possible to generalize the implementation of the intervention to these smaller facilities, the extent to which the PPIUD intervention could be introduced would depend on the level of expertise and capacity among health providers to integrate this additional service into their existing care. To this end, we note that PPIUD insertion services in Sri Lanka are mainly provided by doctors in hospitals, whereas PPIUD insertions in other countries where the FIGO intervention was rolled out are also provided by nurses and other trained medical personnel in hospitals. Findings from these other countries show that PPIUD insertions by nurses and other trained health providers have similar expulsion and complication rates to those by doctors [12], which suggest that insertions need not be limited to only doctors.

## Conclusions

This study demonstrates that incorporating PPIUD services into postpartum maternity care is feasible and potentially effective. Taking the evidence on both counseling and choice of PPIUD together, we find that the intervention had a generally positive impact on receipt of PPIUD counseling and, to a lesser degree, on choice of the PPIUD. Nevertheless, it is clear that the intervention’s effectiveness can be improved to be able to successfully meet the demand for immediate postpartum family planning of women who seek to space or limit births.

## Additional file

Additional file 1: **Table S1.** Intervention timeline, by study hospitals. **Table S2** Descriptive statistics and balance table between study sample at baseline and full study sample. **Table S3.** Difference between baseline and intervention period across each hospital. **Table S4.** PPIUD counseling rates and rates of choice of PPIUD during 3-month baseline period, by hospital. **Table S5.** Difference in Group 1 and Group 2 hospitals during the first 3 months of the study, excluding Nawalapitiya and Nuwara Eliya Hospitals. **Table S6.** Intent-to-treat effect of the intervention on PPIUD counseling, excluding Nawalapitiya and Nuwara Eliya Hospitals. **Table S7.** Intent-to-treat effect of the intervention on choice of PPIUD, excluding Nawalapitiya and Nuwara Eliya Hospitals. **Table S8.** Adherence-adjusted impact of PPIUD counseling on choice of PPIUD—a control function approach, linear probability model, excluding Nawalapitiya and Nuwara Eliya Hospitals. **Figure S1.** Study hospitals. **Figure S2.** Trends in PPIUD counseling rates, excluding Nawalapitiya and Nuwara Eliya Hospitals. **Figure S3.** Trends in choice of PPIUD, excluding Nawalapitiya and Nuwara Eliya Hospitals. (DOCX 519 kb)

## Data Availability

The data that support the findings of this study are available from SLCOG, but restrictions apply to the availability of these data, which were used under license for the current study, and so are not publicly available. Data are, however, available from the authors upon reasonable request and with permission of SLCOG.

## Abbreviations

DHS: Demographic and Health Surveys
FHB: Family Health Bureau
FIGO: International Federation of Gynaecology and Obstetrics
ITT: Intent-to-treat
IUD: Intrauterine device
IV: Instrumental variables
MOH: Ministry of Health
PPFP: Postpartum family planning
PPIUD: Immediate postpartum intrauterine device
SLCOG: Sri Lanka College of Obstetricians and Gynaecologists
WHO: World Health Organization
