# Supplementary material for: The effect of a postpartum IUD intervention on counseling and choice: Evidence from a cluster-randomized stepped-wedge trial in Sri Lanka
Source: Trials. 2019 Jul 8;20:407. doi: 10.1186/s13063-019-3473-6 (PMC6615190; doi:10.1186/s13063-019-3473-6)
Supplement: Supplementary file 1 — Table S1. Intervention timeline, by study hospitals. Table S2 Descriptive statistics and balance table between study sample at baseline and full study sample. Table S3. Difference between baseline and intervention period across each hospital. Table S4. PPIUD counseling rates and rates of choice of PPIUD during 3-month baseline period, by hospital. Table S5. Difference in Group 1 and Group 2 hospitals during the first 3 months of the study, excluding Nawalapitiya and Nuwara Eliya Hospitals. Table S6. Intent-to-treat effect of the intervention on PPIUD counseling, excluding Nawalapitiya and Nuwara Eliya Hospitals. Table S7. Intent-to-treat effect of the intervention on choice of PPIUD, excluding Nawalapitiya and Nuwara Eliya Hospitals. Table S8. Adherence-adjusted impact of PPIUD counseling on choice of PPIUD—a control function approach, linear probability model, excluding Nawalapitiya and Nuwara Eliya Hospitals. Figure S1. Study hospitals. Figure S2. Trends in PPIUD counseling rates, excluding Nawalapitiya and Nuwara Eliya Hospitals. Figure S3. Trends in choice of PPIUD, excluding Nawalapitiya and Nuwara Eliya Hospitals. (DOCX 519 kb) [file 13063_2019_3473_MOESM1_ESM.docx]

### **Table S1: Intervention Timeline, by Study Hospitals**

| **Hospital** | **Group** | **Start Date of Data Collection** | **Planned Start Date of Intervention** | **Date of PPIUD Training** | **Date of First PPIUD Insertion** | **End Date of Data Collection** |
| --- | --- | --- | --- | --- | --- | --- |
| Nawalapitiya | 1 | 7-Sep-15 | 7-Dec-15 | 23-Nov-15 | 7-Sep-15 | 5-Jan-17 |
| Polonnaruwa | 1 | 7-Sep-15 | 7-Dec-15 | 12-Nov-15 | 25-Mar-16 | 5-Jan-17 |
| Chilaw | 1 | 7-Sep-15 | 7-Dec-15 | 1-Dec-15 | 9-Dec-16 | 5-Jan-17 |
| Nuwara Eliya | 2 | 7-Sep-15 | 7-Jun-16 | 29-Jul-16 | 7-Sep-15 | 5-Jan-17 |
| Moneragala | 2 | 7-Sep-15 | 7-Jun-16 | 8-Jul-16 | 16-Jul-16 | 5-Jan-17 |
| Kalutara | 2 | 7-Sep-15 | 7-Jun-16 | 11-Jul-16 | 26-Jul-16 | 5-Jan-17 |

**Table S2: Descriptive Statistics and Balance Table between Study Sample at Baseline and Full Study Sample**

|  | **Full Study Sample** | **Baseline Period** | **Difference^1^** | |
| --- | --- | --- | --- | --- |
| **Panel A** | **n (prop.)** | **n (prop.)** | **Estimate (β)** | **p-value^2^** |
| **Woman's Age**  <20 years | 1984 (0.051) | 797 (0.05) | -0.005 | (0.028) |
| 20-24 years | 9059 (0.231) | 3587 (0.233) | -0.003 | (0.508) |
| 25-29 years | 12692 (0.323) | 5017 (0.326) | 0.013 | (0.148) |
| >=30 years | 15342 (0.395) | 6149 (0.391) | -0.006 | (0.327) |
| **Woman's Schooling**  No Schooling | 291 (0.008) | 128 (0.007) | -0.002 | (0.36) |
| Some Primary | 4338 (0.115) | 1789 (0.108) | 0 | (0.946) |
| Some Lower Secondary | 14657 (0.353) | 5491 (0.39) | 0.029 | (0.313) |
| Some Higher Secondary | 9647 (0.276) | 4289 (0.228) | -0.039 | (0.09) |
| Some College | 10130 (0.248) | 3852 (0.267) | 0.011 | (0.056) |
| **Time taken to travel from home to hospital**  <1 hour | 18362 (0.494) | 6401 (0.511) | -0.02 | (0.193) |
| 1-3 hours | 15981 (0.446) | 5779 (0.436) | 0.01 | (0.292) |
| >=3 hours | 2012 (0.061) | 787 (0.052) | 0.01 | (0.497) |
| **Parity**  1 | 15048 (0.378) | 5869 (0.39) | 0.008 | (0.355) |
| 2 | 14730 (0.374) | 5813 (0.379) | -0.007 | (0.228) |
| 3&+ | 9286 (0.248) | 3859 (0.231) | -0.001 | (0.718) |
| **Ethnicity** Sinhalese | 27685 (0.696) | 10829 (0.716) | 0.002 | (0.755) |
| Sri Lankan Tamil | 4116 (0.102) | 1579 (0.108) | 0.006 | (0.672) |
| Indian Tamil | 3288 (0.104) | 1618 (0.071) | -0.003 | (0.498) |
| Sri Lankan Moor | 3892 (0.094) | 1454 (0.104) | -0.002 | (0.314) |
| Other | 98 (0.005) | 70 (0.001) | -0.003 | (0.149) |
| **Male child born** | 20050 (0.509) | 7918 (0.516) | 0.019 | (0.445) |
| **Received ANC**  Hospital | 18448 (0.468) | 7274 (0.475) | -0.001 | (0.93) |
| MOH Clinic | 31923 (0.753) | 11709 (0.859) | 0.013 | (0.091) |
| **Panel B** |  |  |  |  |
| **Received PPIUD Counselling** | 13648 (0.118) | 1839 (0.502) | See Table 3 | |
| **Ch** **ose PPIUD** | 2736 (0.04) | 619 (0.09) | See Table 4 | |
| **Grand Total** | **39084 (1.000)** | **15552 (1.000)** |  | |

**^1^**The coefficient shown is the coefficient on the post term from equation 1 with variable on each row as dependent variable. The coefficient should be interpreted as the difference between intervention and baseline period, controlling for hospital and month fixed effects.

**^2^**Significance of difference tested using wild cluster bootstrap method

**Table S3: Difference Between Baseline and Intervention Period Across Each Hospital**

|  | **Chilaw** | | | **Kalutara** | | | | **Moneragala** | | | **Nawalapitiya** | | | | **Nuwara Eliya** | | | | **Polonnaruwa** | | |
| --- | --- | --- | --- | --- | --- | --- | --- | --- | --- | --- | --- | --- | --- | --- | --- | --- | --- | --- | --- | --- | --- |
|  | **BL**  **Period** | **Difference [INT-BL]** | **BL**  **Period** | | | **Difference [INT-BL]** | **BL**  **Period** | | **Difference [INT-BL]** | **BL**  **Period** | | | **Difference [INT-BL]** | **BL**  **Period** | | | **Difference [INT-BL]** | **BL**  **Period** | | | **Difference [INT-BL]** |
| **Woman's Age**  <20 years | 0.063 | 0.004 | 0.051 | | | 0.004 | 0.053 | | -0.003 | 0.045 | | | -0.006 | 0.052 | | | -0.004 | 0.04 | | | 0.002 |
| 20-24 years | 0.242 | -0.006 | 0.211 | | | 0.003 | 0.256 | | -0.015 | 0.207 | | | 0.021 | 0.236 | | | -0.01 | 0.228 | | | 0.015 |
| 25-29 years | 0.307 | 0.012 | 0.313 | | | -0.001 | 0.313 | | 0.009 | 0.352 | | | -0.024 | 0.347 | | | -0.005 | 0.311 | | | 0.019 |
| >=30 years | 0.387 | -0.009 | 0.425 | | | -0.006 | 0.378 | | 0.009 | 0.396 | | | 0.006 | 0.365 | | | 0.009 | 0.421 | | | -0.036** |
| **Woman's Schooling** No Schooling | 0.007 | -0.001 | 0.006 | | | 0 | 0.005 | | -0.002 | 0.011 | | | 0.003 | 0.015 | | | -0.005* | 0.005 | | | -0.001 |
| Some Primary | 0.125 | -0.007 | 0.093 | | | -0.006 | 0.08 | | -0.016** | 0.161 | | | -0.027** | 0.176 | | | 0 | 0.085 | | | 0.002 |
| Some Lower Secondary | 0.318 | 0.071*** | 0.383 | | | 0.016 | 0.449 | | 0.043*** | 0.184 | | | 0.121*** | 0.319 | | | 0.048*** | 0.231 | | | 0.171*** |
| Some Higher Secondary | 0.291 | -0.061*** | 0.235 | | | -0.051*** | 0.192 | | -0.044*** | 0.389 | | | -0.087*** | 0.339 | | | -0.072*** | 0.383 | | | -0.158*** |
| Some College | 0.259 | -0.001 | 0.282 | | | 0.04*** | 0.274 | | 0.019* | 0.255 | | | -0.011 | 0.15 | | | 0.029*** | 0.296 | | | -0.015 |
| **Time taken to travel from home to hospital**  <1 hour | 0.71 | -0.035* | 0.686 | | | -0.025** | 0.388 | | 0.056*** | 0.428 | | | -0.04* | 0.279 | | | -0.04*** | 0.555 | | | -0.031* |
| 1-3 hours | 0.272 | 0.031* | 0.284 | | | 0.029*** | 0.544 | | -0.03** | 0.431 | | | 0.064*** | 0.618 | | | 0.042*** | 0.427 | | | 0.016 |
| >=3 hours | 0.018 | 0.005 | 0.03 | | | -0.004 | 0.068 | | -0.026*** | 0.142 | | | -0.024** | 0.103 | | | -0.002 | 0.019 | | | 0.015** |
| **Parity** 1 | 0.409 | -0.017 | 0.353 | | | 0.017* | 0.386 | | -0.002 | 0.357 | | | 0.06*** | 0.403 | | | 0.002 | 0.366 | | | 0.014 |
| 2 | 0.376 | 0.018 | 0.385 | | | -0.02** | 0.387 | | 0.005 | 0.36 | | | -0.008 | 0.342 | | | 0.005 | 0.39 | | | 0.012 |
| 3&+ | 0.216 | -0.001 | 0.262 | | | 0.003 | 0.227 | | -0.003 | 0.283 | | | -0.053*** | 0.255 | | | -0.007 | 0.244 | | | -0.026** |
| **Ethnicity** Sinhalese | 0.846 | -0.017 | 0.767 | | | 0 | 0.935 | | 0.011* | 0.455 | | | -0.024 | 0.3 | | | -0.014 | 0.89 | | | -0.022** |
| Sri Lankan Tamil | 0.06 | 0.002 | 0.043 | | | -0.001 | 0.043 | | -0.009* | 0.271 | | | 0.06*** | 0.243 | | | -0.005 | 0.016 | | | 0.001 |
| Indian Tamil | 0.003 | 0.001 | 0.013 | | | -0.009*** | 0.002 | | 0.001 | 0.174 | | | -0.047*** | 0.385 | | | 0.038*** | 0.001 | | | -0.001 |
| Sri Lankan Moor | 0.088 | 0.016* | 0.175 | | | 0.01 | 0.02 | | -0.004 | 0.099 | | | 0.009 | 0.055 | | | -0.005 | 0.092 | | | 0.021** |
| Other | 0.003 | -0.002 | 0.002 | | | 0 | 0 | | 0.001 | 0.001 | | | 0.002 | 0.016 | | | -0.015*** | 0 | | | 0 |
| **Male child born** | 0.524 | -0.01 | 0.503 | | | 0.011 | 0.514 | | -0.017 | 0.499 | | | 0.023 | 0.515 | | | 0.001 | 0.498 | | | 0.024 |
| **Received ANC**  Hospital | 0.567 | -0.006 | 0.329 | | | -0.015 | 0.548 | | -0.043*** | 0.66 | | | -0.107*** | 0.569 | | | 0.03** | 0.285 | | | 0.104*** |
| MOH Clinic | 0.982 | 0.013*** | 0.311 | | | -0.119*** | 0.94 | | -0.001 | 0.978 | | | 0.011*** | 0.994 | | | 0.001 | 0.871 | | | 0.118*** |
| **N** | **5,779** | | | | **8,625** | | **6,316** | | | | | **4,700** | | | | **6,284** | | | | **7,380** | |
|  |  | | | |  | | **Total N = 39,084** | | | | |  | | | |  | | | |  | |

*** p<0.01, ** p<0.05, * p<0.1

Table S4 PPIUD Counseling Rates and Rates of Choice of PPIUD During 3Month Baseline Period, By Hospital

|  |  | **Mean Rate of PPIUD Counseling by Hospital** | | |  |
| --- | --- | --- | --- | --- | --- |
| **Month** | **Chilaw** | **Kalutara Moneragala Nawalapitiya Nuwara Eliya** | | | **Polonnaruwa** |
| **Sep-15** | 0.011 | 0.013 0.000 0.889 0.293 | | | 0.002 |
| **Oct-15** | 0.002 | 0.013 0.000 0.865 0.227 | | | 0.002 |
| **Nov-15** | 0.009 | 0.022 0.002 0.854 0.273    **Mean Rate of Choice of PPIUD by Hospital** | | | 0.011 |
| **Month** | **Chilaw** | **Kalutara Moneragala Nawalapitiya Nuwara Eliya** | | | **Polonnaruwa** |
| **Sep-15** | 0.000 | 0.002 0.000 | 0.508 | 0.061 | 0.000 |
| **Oct-15** | 0.000 | 0.000 0.000 | 0.473 | 0.059 | 0.000 |
| **Nov-15** | 0.000 | 0.000 0.000 | 0.483 | 0.038 | 0.000 |
| **Total Deliveries in 3-**  **Month Period** | **1155** | **1696 1328** | **913** | **1158** | **1411** |

**Table S5 Difference in Group 1 and Group 2 Hospitals During the First Three Months of the Study – Excluding Nawalapitiya and Nuwara Eliya Hospitals**

|  | **Group 1 Baseline Mean** | **Group 2 Baseline Mean** | **Difference**  **[Group 2 Mean – Group 1 Mean]^1^** |
| --- | --- | --- | --- |
| **Woman's Age**  <20 years | 0.051 | 0.049 | -0.002 |
| 20-24 years | 0.234 | 0.227 | -0.007 |
| 25-29 years | 0.309 | 0.299 | -0.01 |
| >=30 years | 0.406 | 0.426 | 0.019 |
| **Woman's Schooling** No Schooling | 0.006 | 0.006 | 0 |
| Some Primary | 0.103 | 0.103 | 0 |
| Some Lower Secondary | 0.27 | 0.397 | 0.127** |
| Some Higher Secondary | 0.343 | 0.229 | -0.114** |
| Some College | 0.278 | 0.266 | -0.012 |
| **Time taken to travel from home**  **to hospital**  <1 hour | 0.625 | 0.533 | -0.092 |
| 1-3 hours | 0.357 | 0.411 | 0.054 |
| >=3 hours | 0.018 | 0.056 | 0.038 |
| **Parity** 1 | 0.385 | 0.357 | -0.028 |
| 2 | 0.383 | 0.387 | 0.004 |
| 3&+ | 0.232 | 0.255 | 0.024 |
| **Ethnicity** Sinhalese | 0.87 | 0.848 | -0.022 |
| Sri Lankan Tamil | 0.036 | 0.038 | 0.002 |
| Indian Tamil | 0.002 | 0.011 | 0.009 |
| Sri Lankan Moor | 0.09 | 0.102 | 0.012 |
| Other | 0.001 | 0.001 | -0.001 |
| **Male child born** | 0.51 | 0.514 | 0.005 |
| **Received ANC**  Hospital | 0.412 | 0.433 | 0.021 |
| MOH Clinic **Panel B** | 0.921 | 0.64 | -0.281 |
| Received PPIUD Counselling | 0.006 | 0.009 | 0.003 |
| Chose PPIUD | 0 | 0 | 0 |
| **Total N: 5,589** | **2,565** | **3,024** |  |

^1^Significance of difference tested using Wild Cluster Bootstrap method

*** p<0.01, ** p<0.05, * p<0.1

Table S6 **Intent-to-Treat Effect of the Intervention on PPIUD Counselling – Excluding Nawalapitiya and Nuwara Eliya Hospitals**

| Est. | | **Dependent Variable: Counselled on PPIUD** | | | | | | |  |
| --- | --- | --- | --- | --- | --- | --- | --- | --- | --- |
|  |  | 95% CI Est. 95% CI | | | | | | |  |
| **Post-Treatment (Ref: Pre-Treatment)** 0.353*** | | [0.272 - 0.434] | | 0.358*** | | [0.277 - 0.439] | |  |  |
| **Woman's Age (Ref: <20 years)** | |  | |  | |  | |  |  |
| 20-24 years | |  | | 0.022 | | [-0.041 - 0.085] | |  |  |
| 25-29 years | |  | | 0.037 | | [-0.018 - 0.093] | |  |  |
| >=30 years | |  | | 0.024 | | [-0.026 - 0.074] | |  |  |
| **Woman's Schooling (Ref: No schooling)** | |  | |  | |  | |  |  |
| Some Primary | |  | | 0.024 | | [-0.123 - 0.171] | |  |  |
| Some Lower Secondary | |  | | 0.051 | | [-0.073 - 0.176] | |  |  |
| Some Higher Secondary | |  | | 0.066 | | [-0.050 - 0.182] | |  |  |
| Some College | |  | | 0.049 | | [-0.032 - 0.130] | |  |  |
| **Time to travel from home to hospital (Ref: <1 hour)** | |  | |  | |  | |  |  |
| 1-3 hours | |  | | -0.013 | | [-0.055 - 0.030] | |  |  |
| >=3 hours | |  | | -0.074** | | [-0.123 - -0.026] | |  |  |
| **Parity (Ref: 1)** | |  | |  | |  | |  |  |
| 2 | |  | | -0.001 | | [-0.013 - 0.011] | |  |  |
| 3&+ | |  | | 0.003 | | [-0.021 - 0.027] | |  |  |
| **Ethnicity (Ref: Sinhalese)** | |  | |  | |  | |  |  |
| Sri Lankan Tamil | |  | | -0.033 | | [-0.133 - 0.067] | |  |  |
| Indian Tamil | |  | | -0.033** | | [-0.052 - -0.014] | |  |  |
| Sri Lankan Moor | |  | | -0.138** | | [-0.259 - -0.017] | |  |  |
| Other |  | |  | | -0.145 | | [-0.401 - 0.111] | | |
| **Male child born** |  | |  | | -0.007 | | [-0.028 - 0.014] | | |
| Constant | 0.051 | | [-0.058 - 0.161] | | -0.019 | | [-0.153 - 0.116] | | |
| **Observations** | **28,100** | |  | | **26,138** | |  | | |
| **R-squared** | **0.281** | |  | | **0.279** | |  | | |

*** p<0.01, ** p<0.05, * p<0.1

Note: Difference from null tested using wild cluster bootstrap method. All regression models adjusted for hospital and month fixed effects.

### **Table S7: Intent-to-Treat Effect of the Intervention on Choice of PPIUD– Excluding Nawalapitiya and Nuwara Eliya Hospitals**

|  | | **Dependent Variable: Choice of PPIUD** | | |
| --- | --- | --- | --- | --- |
| Est. | | 95% CI | Est. | 95% CI |
| **Post-Treatment (Ref: Pre-Treatment)** 0.025 | | [-0.009 - 0.059] | 0.027 | [-0.011 - 0.064] |
| **Woman's Age (Ref: <20 years)** | |  |  |  |
| 20-24 years | |  | -0.008 | [-0.022 - 0.005] |
| 25-29 years | |  | -0.012* | [-0.028 - 0.003] |
| >=30 years  **Woman's Schooling (Ref: No schooling)** | |  | -0.013 | [-0.034 - 0.008] |
| Some Primary | |  | -0.007 | [-0.023 - 0.010] |
| Some Lower Secondary | |  | -0.009 | [-0.042 - 0.025] |
| Some Higher Secondary | |  | -0.006 | [-0.033 - 0.021] |
| Some College | |  | -0.006 | [-0.042 - 0.029] |
| **Time to travel from home to hospital (Ref: <1 hour)** | |  |  |  |
| 1-3 hours | |  | -0.001 | [-0.009 - 0.007] |
| >=3 hours | |  | -0.006 | [-0.017 - 0.004] |
| **Parity (Ref: 1)** | |  |  |  |
| 2 | |  | 0.011** | [0.001 - 0.021] |
| 3&+ | |  | 0.008 | [-0.008 - 0.024] |
| **Ethnicity (Ref: Sinhalese)** | |  |  |  |
| Sri Lankan Tamil | |  | -0.007* | [-0.015 - 0.001] |
| Indian Tamil | |  | -0.022*** | [-0.032 - -0.011] |
| Sri Lankan Moor | |  | -0.012* | [-0.027 - 0.002] |
| Other | |  | -0.023*** | [-0.027 - -0.020] |
| **Male child born** | |  | -0.001 | [-0.007 - 0.004] |
| Constant | 0.005 | [-0.022 - 0.032] | 0.015 | [-0.022 - 0.053] |
| **Observations** | **28,100** |  | **26,138** |  |
| **R-squared** | **0.025** |  | **0.027** |  |

*** p<0.01, ** p<0.05, * p<0.1

Note: Difference from null tested using wild cluster bootstrap method. All regression models adjusted for hospital and month fixed effects.

### **Table S8: Adherence-Adjusted Impact of PPIUD Counselling on Choice of PPIUD– A Control Function Approach, Linear Probability Model, Excluding Nawalapitiya and Nuwara Eliya Hospitals**

| Est. | | | | | **Choice of PPIUD** | | | | |  |
| --- | --- | --- | --- | --- | --- | --- | --- | --- | --- | --- |
|  |  |  |  |  | 95% CI Est. | | | | | 95% CI |
| **Counselled on PPIUD** 0.071* [-0.014 - 0.157] | | | | | | 0.075* | | [-0.018 - 0.168] | | |
| **Woman's Age (Ref: <20)** | | | | |  |  | |  | | |
| 20-24 years | | | | |  | -0.010* | | [-0.023 - 0.003] | | |
| 25-29 years | | | | |  | -0.015* | | [-0.030 - 0.000] | | |
| >=30 years | | | | |  | -0.015 | | [-0.035 - 0.006] | | |
| **Woman's Schooling (Ref: No schooling)** | | | | |  |  | |  | | |
| Some Primary | | | | |  | -0.008 | | [-0.032 - 0.015] | | |
| Some Lower Secondary | | | | |  | -0.013 | | [-0.052 - 0.027] | | |
| Some Higher Secondary | | | | |  | -0.011 | | [-0.043 - 0.021] | | |
| Some College | | | | |  | -0.010 | | [-0.049 - 0.028] | | |
| **Time to travel from home to hospital (Ref: <1 hour)** | | | | |  |  | |  | | |
| 1-3 hours |  | |  | |  | -0.000 | | [-0.006 - 0.006] | | |
| >=3 hours |  | |  | |  | -0.001 | | [-0.012 - 0.010] | | |
| **Parity (Ref: 1)** |  | |  | |  |  | |  | | |
| 2 |  | |  | |  | 0.011** | | [0.001 - 0.021] | | |
| 3&+ |  | |  | |  | 0.008 | | [-0.008 - 0.024] | | |
| **Ethnicity (Ref: Sinhalese)** |  | |  | |  |  | |  | | |
| Sri Lankan Tamil |  | |  | |  | -0.005 | | [-0.017 - 0.008] | | |
| Indian Tamil |  | |  | |  | -0.019*** | | [-0.030 - -0.009] | | |
| Sri Lankan Moor |  | |  | |  | -0.002 | | [-0.020 - 0.016] | | |
| Other |  | |  | |  | -0.012 | | [-0.032 - 0.007] | | |
| **Male child born** |  | |  | |  | -0.001 | | [-0.006 - 0.005] | | |
| **Control Function (FS Resids)** | -0.017 | | [-0.084 - 0.050] | | | -0.021 | | [-0.097 - 0.055] | | |
| Constant | 0.001 | | [-0.024 - 0.027] | | | 0.017 | | [-0.023 - 0.057] | | |
|  |  | |  | | |  | |  | | |
| **Observations** | | **28,100** | | | | | **26,138** | | | |
| **R-squared** | | **0.051** | | | | | **0.052** | | | |

*** p<0.01, ** p<0.05, * p<0.1

Note: Difference from the null hypothesis are tested using the wild-cluster bootstrap method. Second stage results are shown above; first stage results are presented in **Appendix Table 6**. All regression models adjusted for hospital and month fixed effects.


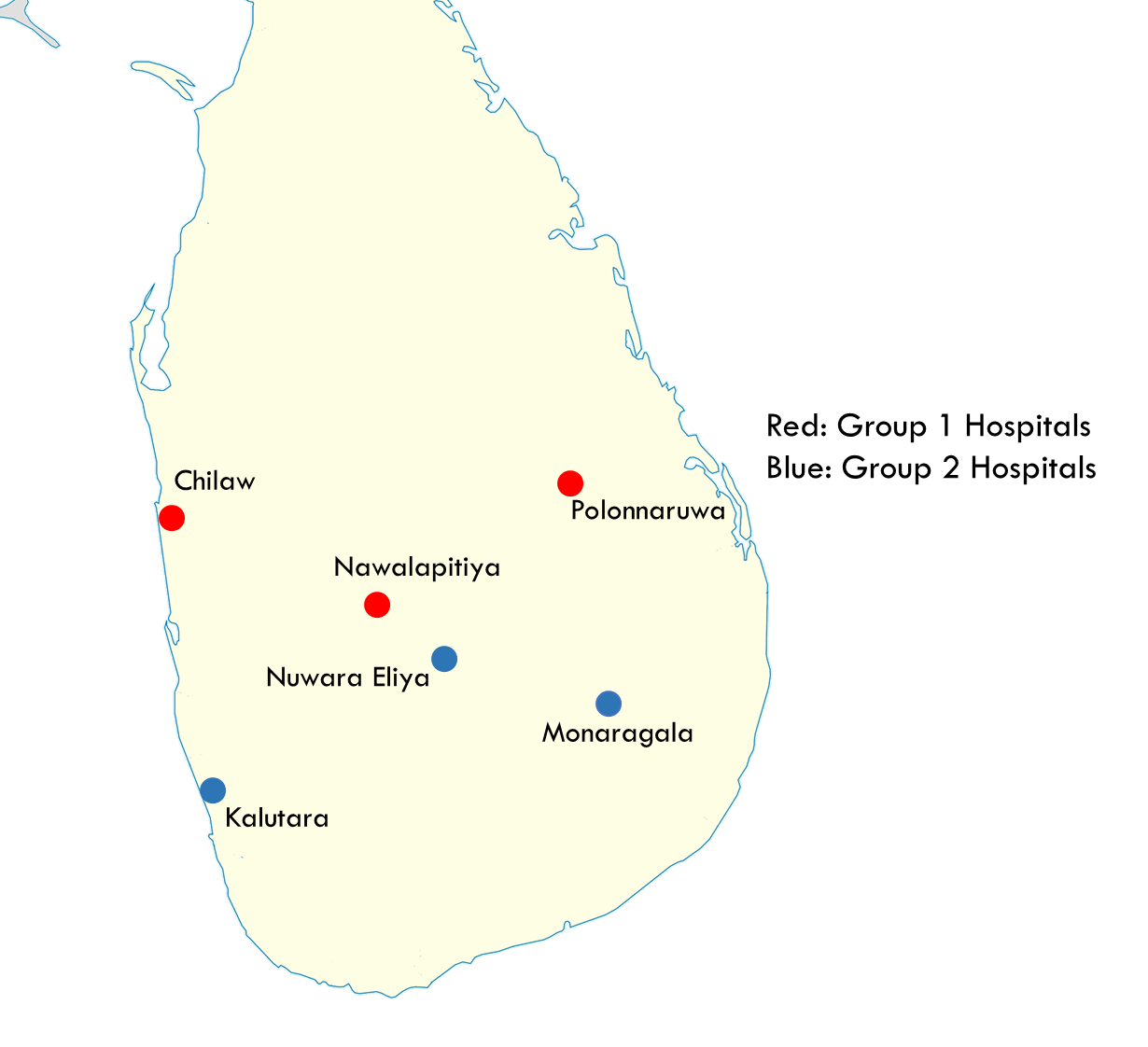


**Figure S1: Study Hospitals**


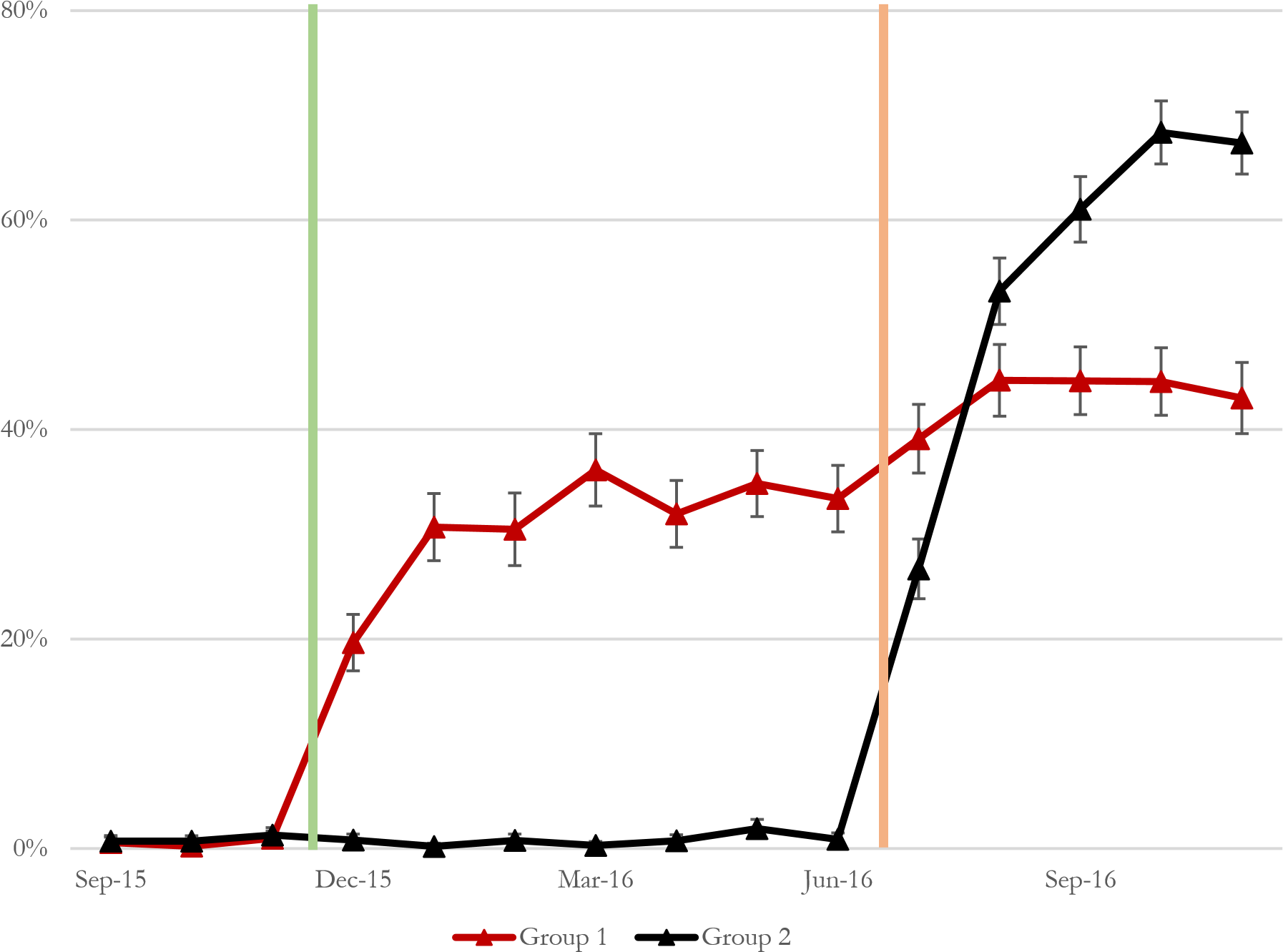


PPIUD Counseling Rate

### **Figure S2: Trends in PPIUD Counselling Rates, Excluding Nawalapitiya and Nuwara Eliya Hospitals**

Note: Standard errors shown as error bars. Approximate intervention start dates in Group 1 (red) and Group 2 (black) hospitals are shown by the green and orange vertical lines, respectively. For exact dates of intervention, please see **Additional file 1: Table S1**.


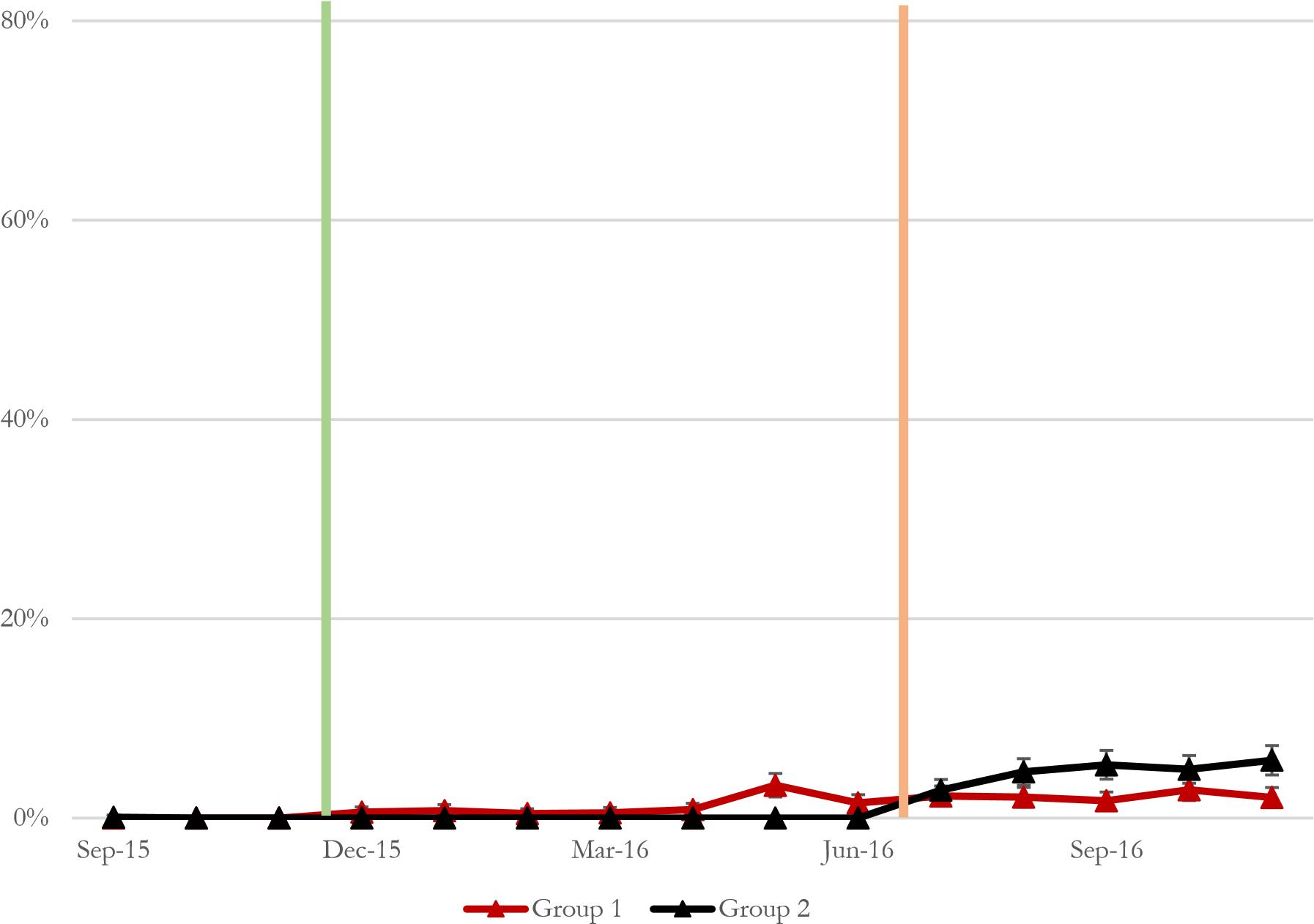


Proportion who chose PPIUD

### **Figure S3: Trends in Choice of PPIUD, Excluding Nawalapitiya and Nuwara Eliya Hospitals**

Note: Standard errors shown as error bars. Approximate intervention start dates in Group 1 (red) and Group 2 (black) hospitals are shown by the green and orange vertical lines, respectively. For exact dates of intervention, please see **Additional file 1: Table S1**.
